# Shift of Microbiota and Modulation of Resistome in the Ceca of Broiler Chicken Fed Berry Pomace Alone or in Combination of a Multienzyme Mixture

**DOI:** 10.3390/microorganisms13051044

**Published:** 2025-04-30

**Authors:** Munene Kithama, Yousef I. Hassan, Xianhua Yin, Joshua Tang, Lindsey Clairmont, Olimpia Sienkiewicz, Kelly Ross, Calvin Ho-Fung Lau, Dion Lepp, Xin Zhao, Elijah G. Kiarie, Moussa S. Diarra

**Affiliations:** 1 Guelph Research and Development Centre, Agriculture and Agri-Food Canada (AAFC), Guelph, ON N1G 5C9, Canada; m.kithama@pu.ac.ke (M.K.); joshua.tang@agr.gc.ca (J.T.); lindsey.clairmont@agr.gc.ca (L.C.); dion.lepp@agr.gc.ca (D.L.); 2 Department of Animal Biosciences, University of Guelph, Guelph, ON N1G 2W1, Canada; 3 Department of Animal Science, McGill University, Montreal, QC H9X 3V9, Canada; 12ohs@queensu.ca (O.S.);; 4 Summerland Research and Development Centre, Agriculture and Agri-Food Canada (AAFC), Summerland, BC V0H 1Z0, Canada; 5 Ottawa Laboratory (Carling), Canadian Food Inspection Agency, Ottawa, ON K1A 0C6, Canada

**Keywords:** broiler chicken, ceca, berry pomace, microbiota, antimicrobial resistance

## Abstract

Alternative feed additives are being investigated due to the restriction of antibiotics use to decrease antimicrobial resistance (AMR) in food-producing animals. This study investigated the effects of dietary American cranberry (*Vaccinium macrocarpon*) and wild blueberry (*V. angustifolium*) pomaces on the cecal microbiota and resistome profiles as well as the short-chain fatty acid levels. Male broiler chickens Cobb500 were fed a basal diet with either 55 ppm bacitracin methylene disalicylate (BMD); 0.5% (CRP0.5) and 1% (CRP1) cranberry pomace; and 0.5% (LBP0.5) and 1% (LBP1) lowbush blueberry pomace with or without a multienzyme mixture (ENZ). The results showed that at 21 days of age, the total coliform counts decreased in the CRP0.5-fed birds compared to BMD (*p* < 0.05). The use of pomace significantly increased the abundance of *Lactobacillus* and *Bacteroides* regardless of ENZ, while CRP decreased the Proteobacteria phylum abundance. In-feed ENZ tended to increase the relative abundance of genes conferring aminoglycoside resistance. Treatment with CRP0.5 decreased the abundance of *cepA* genes encoding for macrolide (*MACROLIDE)* and lincomycin (*InuD*) resistance while increasing those for tetracycline (*tetO* and *tetX*) resistance. These results showed, for the first time, the potential of the studied enzymes in influencing berry pomace’s effects on antimicrobial resistance gene profiles in broilers.

## 1. Introduction

The poultry gut microbiota community structure, diversity, and function have been the subject of studies, with progressive improvement in techniques to identify different taxonomic units [1]. However, both culture-dependent and -independent techniques are still deployed even though less than 20% of the gastrointestinal bacteria have been cultured due to the fact that most bacteria are fastidious and their growing environments both in vivo and in vitro are unknown [1]. These two tools (culture-dependent and -independent) have, nonetheless, assisted researchers in obtaining insights into the microbial ecology of humans and animals including poultry as described below.

Chicken gut microbiota is affected by various factors, including age, housing, environmental conditions, and diet, which play major roles in influencing microbial population dynamics [1,2]. Extensive research has shown that three to four bacterial phyla including Firmicutes, Bacteroidetes, Proteobacteria, and Actinobacteria predominate the chicken gut from the embryonic stage to adult age [3]. The abundance of these bacterial populations varies from one region of the intestine to another, playing specific individual roles that make each region of the gut stand out as an ecosystem of its own [4]. Gut bacterial species are involved in various functions such as nutrient exchange, immunomodulation, competitive exclusion of pathogenic bacteria, and physiological modification of the gut, especially in chicks [5].

Antibiotics have been used in poultry and livestock productions for decades to improve growth performance and for infectious disease prevention which involve the modulation of the gut microbiota. However, the overuse and misuse of antibiotics in food production contributed to the spread of antimicrobial resistance (AMR) in the food chain [6,7]. Commensal bacteria in the gut such as *Escherichia coli* (*E. coli*) can acquire antimicrobial-resistant genes (ARGs) from other bacterial species. In the same ecological niche, such resistant strains can further transmit their ARGs to pathogens which could cause disease in both humans and animals [8]. Research by Winokur et al. [9] demonstrated that *Salmonella* and *E. coli* isolates from cattle, swine, and human samples harbored an identical plasmid-mediated AmpC-like beta-lactamase gene (*CMY-2*) that confers resistance to third-generation cephalosporins such as cephamycin. Southern blot analyses of these isolates showed conserved fragments among many of the *CMY-2*-plasmids indicating possible gene transfer between these bacterial species. Monitoring ARGs in the broiler industry is increasing due to a combination of factors including consumers’ demands for safe and healthy food, and increasing pressure from government agencies on the poultry industry to seek alternatives to in-feed antibiotics [10]. Accordingly, the use of gentamicin and ceftiofur to control avian colibacillosis exerted selection pressures for resistance against these antibiotics to such an extent that even after a stoppage of their use, co-selection of gentamicin resistance was still apparent when spectinomycin and lincomycin were used [11].

Technological advancements have made it easier and less expensive to understand and monitor the emergence, spread, and sustenance of AMR and their related ARGs in the environment [12]. Some concepts such as mutator state have shed light on integrative conjugative elements and insertion sequences with common region (ISCR) elements, and discoveries of mutant selection windows for resistances that come about through mutations in existing genes. These concepts, together with omics studies (metagenomics, transcriptomics, proteomics, and metabolomics), enable the understanding of the epidemiology of carriers of resistant genes and equip scientists, governments, industry players, and livestock producers to monitor resistome (ensemble of ARGs in a microbiota) in broilers [12,13]. Among broiler and swine farmers, resistance to extended-spectrum cephalosporins in *Salmonella enterica* serovar Heidelberg has been recorded [14,15] as well as resistance to the same antimicrobial in *E. coli* from surface waters of dairy farms [16]. For the latter case of dairy farms, researchers showed a phylogenetic and genomic similarity between the *E. coli* they sequenced [16].

Dietary interventions are being used by broiler chicken producers to improve growth and prevent enteric pathogens [17]. Human nutrition research has paved the way in investigating the beneficial effects of dietary interventions, especially from phytochemicals, on the gut microbiota and the subsequent amelioration of such conditions as colitis and obesity [18]. Feed additives and/or ingredients such as probiotics, prebiotics, commercial phytogenic products, fruit pomaces, and exogenous enzymes are some of the products that have been investigated in human as well as in swine and broiler diets and shown to have marked advantages in improving growth and modulating gut bacteria populations [19,20,21,22,23,24,25,26,27]. Metabolites such as ammonia and putrefactive short-chain fatty acids (SCFAs) concentrations were significantly reduced when turkey poults were fed with diets supplemented with dried fruit pomaces of apple, strawberry, and black currant [22].

The present study is the continuation of our research efforts on fruit pomaces in developing cost-effective alternative strategies to improve/maintain broiler health, productivity, and safety. Previous results from analyses of samples derived from experimental trials of this research showed interesting dynamics that occur biochemically and physiologically in birds fed the berry diets [28]. This present study, thus, further evaluated the effects of dietary cranberry and blueberry pomaces, without or with a multienzyme mixture on cecal microflora, resistome, and metabolite profile.

## 2. Materials and Methods

### 2.1. Birds Housing, Diets and Experimental Design

A total of 3150-day-old Cobb 500 chicks (Agri-Marche, St. Isidore, QC, Canada) were placed in a 70-floor pens (45 birds/pen) barn which were allocated to 10 treatments (7 pens/treatment) using a complete randomized block design (Centre de recherché en sciences animales de Deschambault, CRSAD, Deschambault, QC, Canada) and raised as previously described [29]. All the experimental procedures performed in this study were approved by the Animal Care Committee of the CRSAD (protocol # 1920-AV-397, Deschambault) according to the guidelines described by the Canadian Council on Animal Care [30]. Details of the starter (days 1–14), grower (14–28), and finisher (28–35) corn–soybean meal-based diets formulated to meet the nutritional requirements have been described previously by Kithama et al. [29]. Briefly, the diets consisted of a feed with bacitracin methylene disalicylate (BMD) at 55 mg/kg, and low bush blueberry pomace (LBP/BLP) and cranberry pomace (CRP) at 0.5% and 1% inclusion levels. Each diet was fed with or without multienzyme supplement, containing galactanase, protease, β-mannanase, β-glucanase, xylanase, α-amylase, and cellulase activities at 50, 200, 400, 600, 7000, 2500, and 2800 U/g of product, respectively (Canadian Bio-systems Inc., Calgary, AB, Canada).

### 2.2. Sample Collections

At 21 (D21) and 35 (D35) days of age, 14 birds/treatments (2 birds/pen close to average pen body weight) were euthanized (*n* = 280 birds; 140 birds/sampling day). Cecal contents were aseptically collected from each bird and transferred to sterile Whirl-Pak plastic bags for culture-dependent and -independent microbiota analyses as previously described (19). DNA samples from the cecal digesta were extracted for bacterial 16S rRNA sequencing, targeted resistome, and some metabolites analysis. The samples were stored at −80 °C until processing.

### 2.3. Bacterial Enumeration

The cecal samples for bacterial culture were processed according to modified methods described by Taggar et al. [16]. Briefly, 1 g of cecal sample was mixed in 4 mL of 0.85% saline and vortexed to homogenize. Serial 10-fold dilutions (1:10) of the samples in saline were spread on MacConkey Agar (MCA) plates. The plates were incubated at 37 °C for 18–24 h and the colony was counted using a colony counter (Reichert Darkfield Quebec colony counter, ThermoFisher Scientific, Mississauga, ON, Canada). The bacterial numbers were reported as CFU/mL and the values log-transformed for statistical analyses.

### 2.4. DNA Extraction, 16S rRNA Sequencing and Targeting Resistome

Genomic DNA of cecal bacteria for 16S rRNA sequencing was extracted using the DNeasy^®^ PowerSoil^®^ kits (Qiagen, Toronto, ON, Canada) according to the manufacturer’s instruction. DNA quality was analyzed as described by Das et al. Sequencing libraries of the 16S rRNA gene were prepared according to the Illumina 16S Metagenomic Sequencing Library Preparation Guide as detailed by Das et al. [19]. Briefly, the primers Bakt_341F (5′-CCTACGGGNGGCWGCAG-3′) and Bakt_805R (5′-GACTACHVGGGTATCTAATCC-3′) containing 5′ Illumina overhang adapter sequences (5′ TCGTCGGCAGCGTCAGATGTGTATAAGAGACAG and 5′ CTCGTGGGCTCGGAGATGTGTATAAGAGACAG, respectively) were used to amplify an ~537 bp fragment of the 16S rRNA V3-4 region. Each reaction containing 12.5 ng template DNA, 200 nM each primer, and 1 × KAPA HiFi HotStart ReadyMix in a 25 μL volume was amplified using the following cycling conditions: 95 °C for 3 min, 25 cycles of 95 °C for 30 s, 55 °C for 30 s, and 72 °C for 30 s, followed by 72 °C for 5 min. The PCR products were purified with Ampure XP beads (Beckman Coulter, Mississauga, ON, Canada) and sequencing adapters containing 8 bp indices were added to the 3′ and 5′ ends by PCR using the Nextera XT Set A and D Index kits (Illumina, San Diego, CA, USA) in a 50 μL reaction containing 5 μL PCR amplicon, 5 μL each indexing primer, and 25 μL 2× KAPA HiFi HotStart ReadyMix using the following cycling conditions: 95 °C for 3 min, 8 cycles of 95 °C for 30 s, 55 °C for 30 s, and 72 °C for 30 s, followed by 72 °C for 5 min. Following purification with Ampure XP beads, the amplicons were quantified using the Quant-iT PicoGreen double-stranded DNA assay (Invitrogen, Mississauga, ON, Canada), and pooled at equimolar ratios to a final concentration of 8 pM, combined with 10% equimolar PhiX DNA (Illumina), and sequenced on a MiSeq instrument using the MiSeq 600-cycle v3 kit (Illumina).

The bacterial diversity analysis was performed with Quantitative Insights Into Microbial Ecology (QIIME 2) [31]. Briefly, 300 bp paired-end reads were processed with DADA2, which is based on sequencing error correction algorithms and resolves very accurately variants that differ even by a single nucleotide [32], to denoise reads and to remove chimeric sequences and singletons, with paired-ends and de-replicate sequences joined to produce unique amplicon sequence variants (ASVs). The taxonomic classification of the resulting feature table was performed with VSEARCH [33] and the Greengenes 99% OTU sequences [34] as references. Prodan et al. (2020) showed that ASV approaches, in particular DADA2, lead to spurious ASVs in both defined mock communities and real-world samples [35]. To remove spurious ASVs, those with extremely low abundance (fewer than 10 instances across all the samples or present in fewer than two samples), or not assigned taxonomy at the phylum level, were discarded. Multiple sequence alignment of ASV representative sequences was performed with MAFFT [36] and a rooted phylogenetic tree was constructed with FastTree [37]. A core diversities analysis was performed using a sampling depth of 7000 sequences to plot taxonomic relative abundances, calculate alpha-diversity metrics, and generate dissimilarity matrices based on Bray–Curtis and UniFrac distances, which were used for principal coordinate (PCoA) analyses. Heatmaps and PCoA plots were generated with R and PhyloToast [38], respectively. An analysis of composition of microbiomes (ANCOM BC) was used to determine differences in taxa relative abundances between the treatment groups, accounting for the compositional nature of relative abundance data [39]. Songbird [40] was also used to calculate log-fold differentials for each ASV between treatment groups by multinomial regression, and rankings were visualized with Qurro [41].

A total of 60 (pooled of two birds/pen, six pens/treatment) cecal DNA samples from 35-day-old broilers were investigated for ARGs using targeted resistome. A target-enrichment sequencing approach utilizing a myBaits^®^ Custom DNA-Seq kits was used as previously described by Shay et al. [42].

### 2.5. Nuclear Magnetic Resonance (NMR) Analysis

The cecal samples collected at D21 and D35 were analyzed by NMR for butyrate, glutamate, valerate, and propionate profiles [43,44] at the Drug Discovery Platform, Research Institute of the McGill University Health Center (Montreal, QC, Canada). Spectral processing (Fourier transformation, phase correction, baseline correction, and calibration) was performed automatically using the TopSpin 4.4 software, integration calculations were performed in TopSpin 4.4, and quantification and curve fitting were performed in Chenomx NMR Suite 9.0.

### 2.6. Statistical Analyses

Statistical analyses on the relative abundance of coliform counts and abundance of antimicrobial resistance genes were conducted according to a complete randomized block design using the General Linear Mixed Model (GLMM) procedure of the Statistical Analysis System (SAS) version 9.4 (SAS Institute Inc., 2016, Cary, NC, USA) [45]. Statistically significant differences in the resistome alpha-diversity data were determined by the Kruskal–Wallis ANOVA test while Beta-diversity was determined through principle coordinates analysis (PCoA) of Bray–Curtis dissimilarity, both weighted and unweighted Unifrac [42]. The sources of variations were dietary treatments (basal diet + BMD as the control and basal diet + CRP, LBP alone or in combination) and ENZ as well as their interactions (ENZ*Treat) using pens as experimental units (7 pens/treatment groups). Least significance difference (LSD) was used to separate treatment means whenever the *F*-value was significant. The differential abundances of individual taxa between groups associated with different diets were determined using the analysis of composition of microbiomes with Bias Correction (ANCOM-BC2) methodology [46]. Differential abundance based on the mixed-directional false discovery rate-controlled Dunnett’s type of test output of ANCOM-BC2 was reported as log-fold changes (Log FCs) with standard errors relative to the reference group fed the BMD diet without the multienzyme mixture. The association between resistome and bacterial taxa was determined by Spearman’s correlation procedures of SAS. A *p*-value of ≤0.05 was used to declare significance, while a *p*-value of >0.05 but <0.08 was used to declare tendency. Data for the NMR analysis of the fecal samples was analyzed under a General Linear Mixed Model using univariate analysis for both sampling time points combined. To compare time points individually, a one-way ANOVA and Welch’s test were performed for significance.

## 3. Results

### 3.1. Bacterial Counts

The viable counts of coliforms revealed interactions between the ENZ and LBP treatment of D21 coliform numbers (*p* = 0.054), while significant differences were noted between treatments (diets) (*p* = 0.003) on D35 coliform counts, with counts being lower in the CRP0.5-fed birds (Table 1). At the same D35, the use of ENZ in feed decreased the coliform numbers (*p* = 0.005).

### 3.2. Ceca Bacterial Diversity

**Figure 1:**
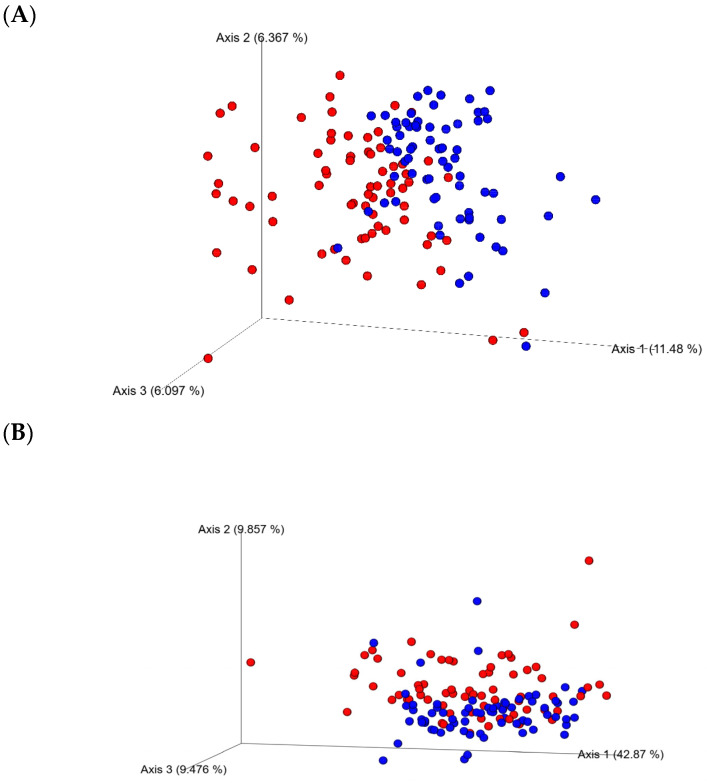
Beta-diversity of bacterial communities in the ceca from the broiler chicken fed bacitracin, berry pomace (studied) diets alone or in combination with a multienzyme mixture. The principal coordinate analysis (PCoA) plots of unweighted (**A**) and weighted (**B**) Unifrac distance matrices, colored by day: (Red) 21-day-old and (Blue) 35-day-old. Significant clustering of samples from both days (PERMANOVA, *p* < 0.05) is shown.

The analysis of the 16S rRNA sequences using the Kruskal–Wallis test of the two collection time (day 21 and 35 of age) samples showed sampling days’ effects on alpha-diversity as showed by the Evenness, Chao, Simpson, and Shannon indices (*p* < 0.0001). No significant differences between enzyme (yes or no) or diets for alpha-diversity indices at any of the sampling days 21 or 35 were found (*p* > 0.05). However, a tendency to increase the richness of cecal microbiota in the CRP0.5-treated birds was observed. Beta-diversity, as estimated by PERMANOVA for unweighted and weighted Unifrac group significance, demonstrated dissimilarities (*p* < 0.01) between the ceca bacterial communities from D21 and D35 (Figure 1).

**Figure 2:**
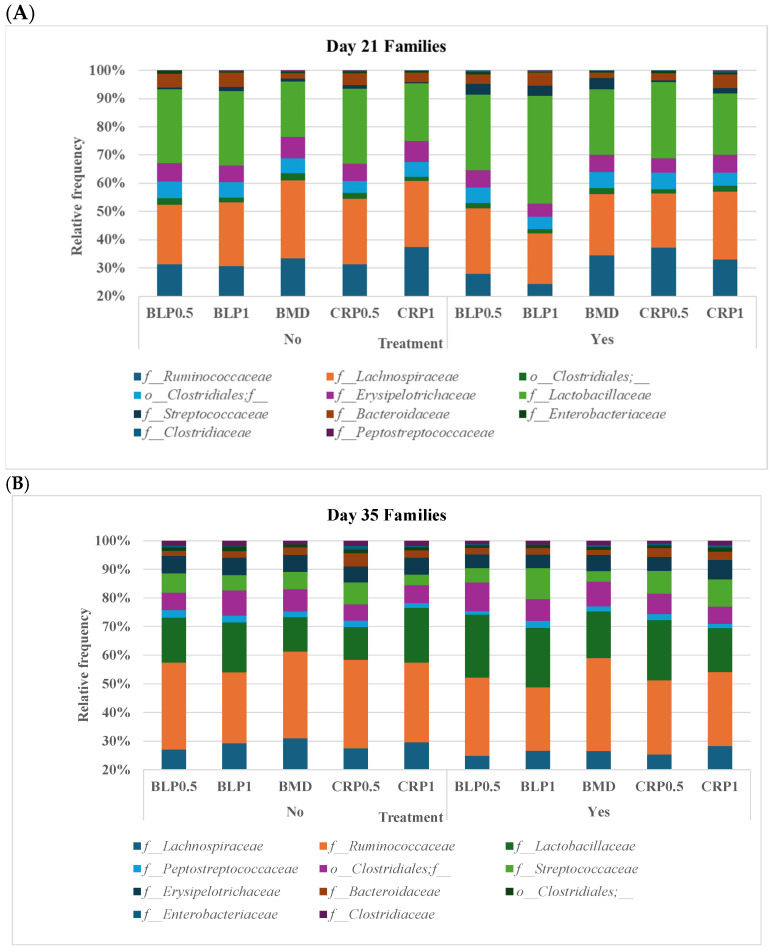
Taxonomic profile of bacterial communities at the family level in the cecal samples of 21- (**A**) and 35- (**B**) day-old broiler chickens fed bacitracin (BMD), or cranberry (CRP at 0.5 and 1%) and blueberry (LBP at 0.5 and 1%) pomaces alone (No) or in combination (Yes) with a multienzyme supplement. (*n* = seven pens/treatment, 2 birds/pen: 14 birds/treatment).

**Figure 3:**
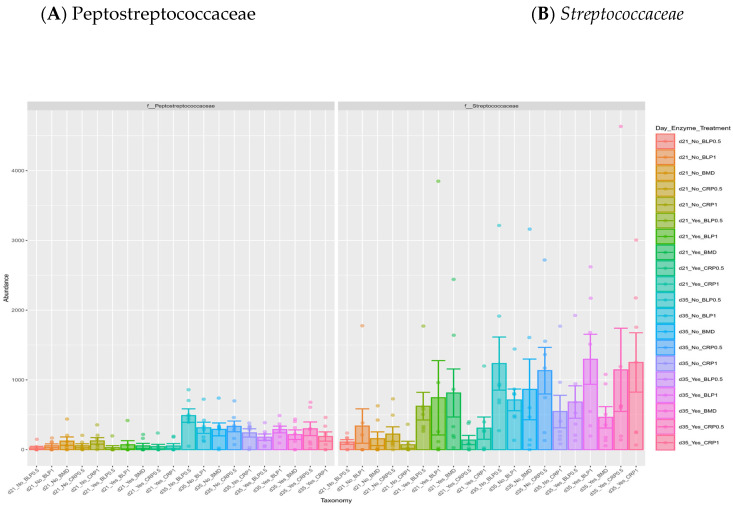
Relative abundances of cecal *Peptostreptococcaceae* (**A**) and *Streptococcaceae* (**B**) families: (**A**) significant differences between 21- and 35-day-old broiler chickens fed bacitracin (BMD), or cranberry (CRP at 0.5 and 1%) and blueberry (LBP at 0.5 and 1%) pomaces alone (No) or in combination (Yes) with a multienzyme mixture. (*p* < 0.05).

At both D21 and D35, Firmicutes, Bacteroidetes, and Proteobacteria were the most abundant bacterial phyla, while 11 families including *Ruminococcaceae*, *Lachnospiraceae*, *Erysipelotrichaceae*, *Lactobacillaceae*, *Streptococcaceae*, *Bacteroidaceae*, *Enterobacteriaceae*, *Clostridiaceae*, and *Peptostreptococcaceae* were common at both sampling days but at different proportions (Figure 2). At day 35, significant differences (*p* < 0.05) were observed between days 21 and 35 for *Peptostreptococcaceae* and *Streptococcaceae* abundances regardless of enzyme use (Figure 3).

Differential abundance analyses of composition using ANCOM-BC were performed to identify bacterial taxa showing absolute abundance impacted by the diets relative to the No-ENZ-BMD control diet as reference.

**Figure 4:**
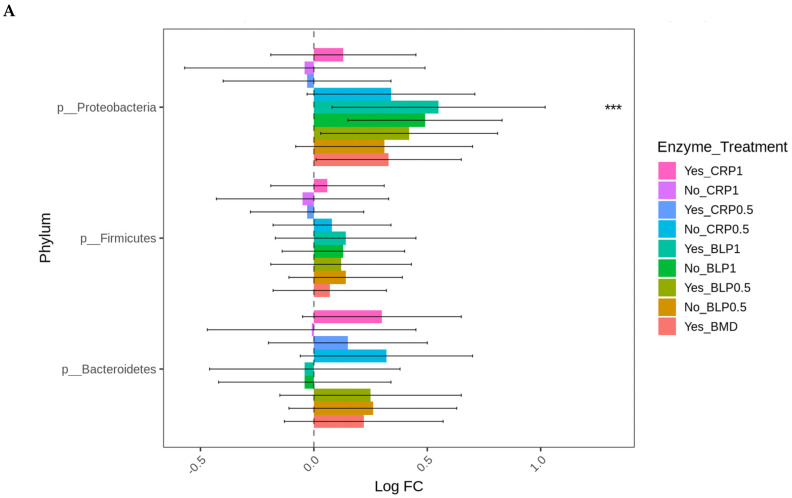

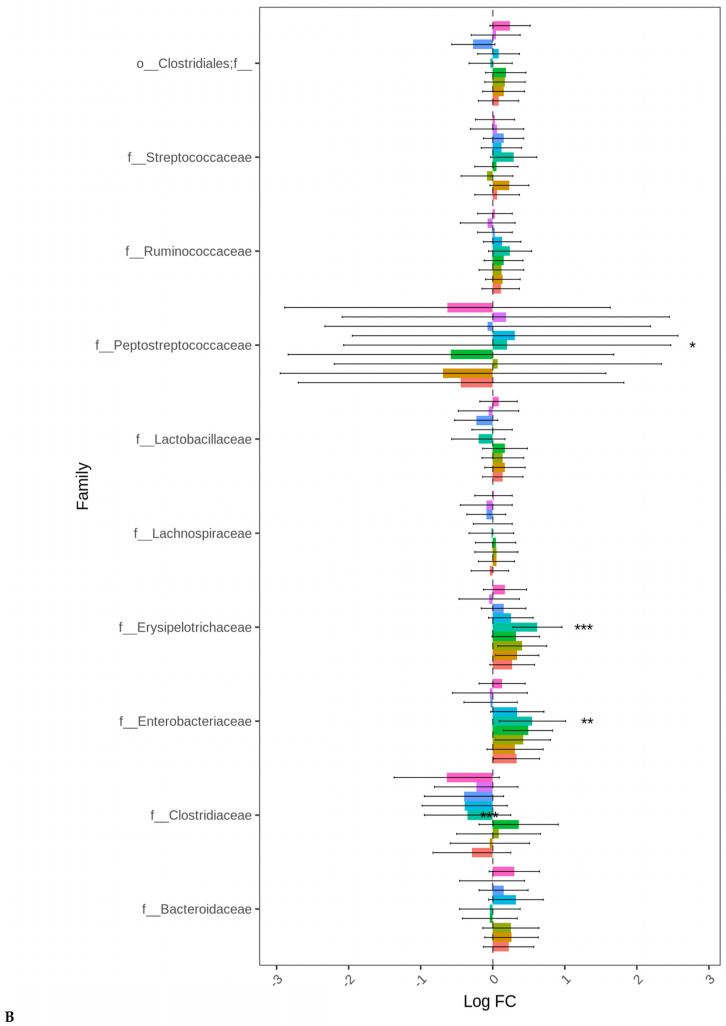
Cecal bacterial phylum (**A**) and family (**B**) of day 21 of age broiler chickens were significantly affected by the dietary bacitracin (BMD) or cranberry (CRP at 0.5 and 1%) and blueberry (LBP at 0.5 and 1%) pomaces alone (No) or in combination (Yes) with a multienzyme mixture. The horizontal axis represents log-fold changes in absolute abundance associated with the diet with or without enzyme (enzyme = treatment) relative to the BMD alone (reference) as determined by the differential abundance analysis performed using ANCOM-BC2. Taxa with statistically significant changes in their abundance relative to the reference group are shown (* *p* < 0.05, ** *p* < 0.01, and *** *p* < 0.001); the bars represent standard errors.

At D21, the highest log-fold change (Log FC) in abundant Proteobacteria was found in the ceca of the birds fed LBP1 regardless of enzyme and LBP0.5 + ENZ while the lowest Log FC abundances of this phylum (Proteobacteria) were observed in the birds receiving CRP1 or CRP0.5 + ENZ and CRP1 alone (*p* < 0.001). Except for the birds fed CRP1 with or without ENZ and CRP0.5 + ENZ, *Erysipelotrichaceae* (*p* < 0.01) and *Enterobacteriaceae* (*p* < 0.01) families were abundant in all the other treatment groups with the birds fed LBP + ENZ showing the highest Log FC of these family abundances (Figure 4A,B).

**Figure 5:**
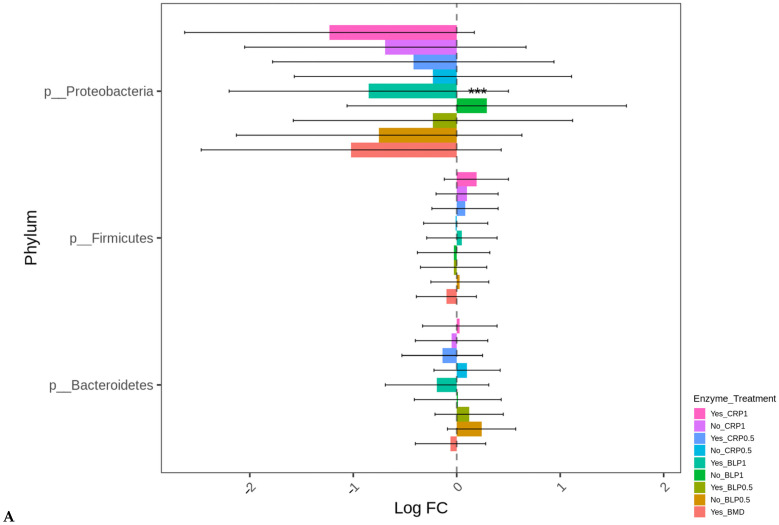

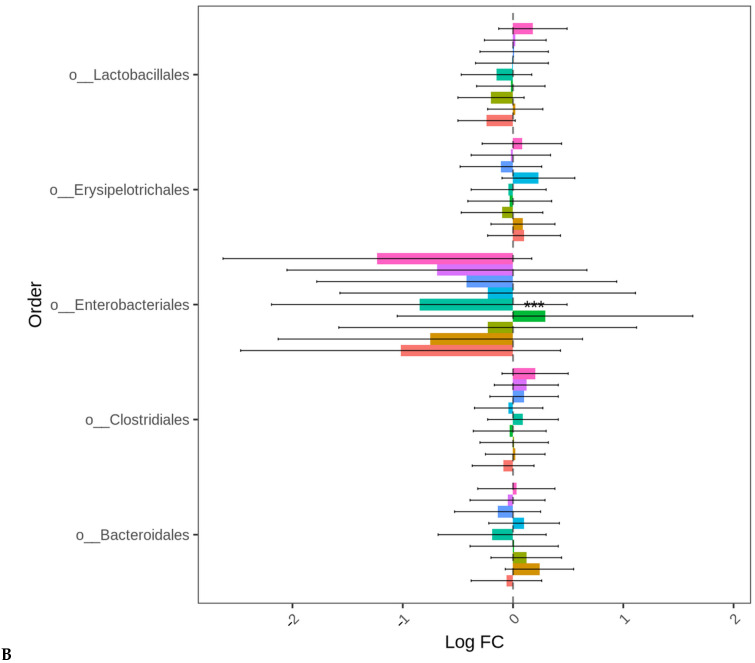
Cecal bacterial phylum (**A**) and order (**B**) of day 35 of age broiler chickens significantly affected by the dietary bacitracin (BMD) or cranberry (CRP at 0.5 and 1%) and blueberry (LBP at 0.5 and 1%) pomaces alone (No) or in combination (Yes) with a multienzyme mixture. The horizontal axis represents log-fold changes in absolute abundance associated with the diet with or without enzyme (enzyme = treatment) relative to the BMD alone (reference) as determined by the differential abundance analysis performed using ANCOM-BC2. Taxa with statistically significant changes in their abundance relative to the reference group are shown (*** *p* < 0.001); the bars represent standard errors.

At D35, except for the birds fed LBP1 alone, the abundance of Proteobacteria decreased in all the other groups (*p* < 0.001). No significant differences in the differential abundance of Firmicutes and Bacteroidetes were observed for any treatment group compared to BMD alone as the control. The order *Enterobacteriales* presented similar differential abundance change profiles (*p* < 0.001) as those of the Proteobacteria indicating that the major change in this phylum was the order *Enterobacteriales* (Figure 5A,B). No significant differences in the absolute abundances of other taxa levels were observed for any treatment groups compared to BMD alone as the control.

### 3.3. Targeted Resistome Profile

**Figure 6:**
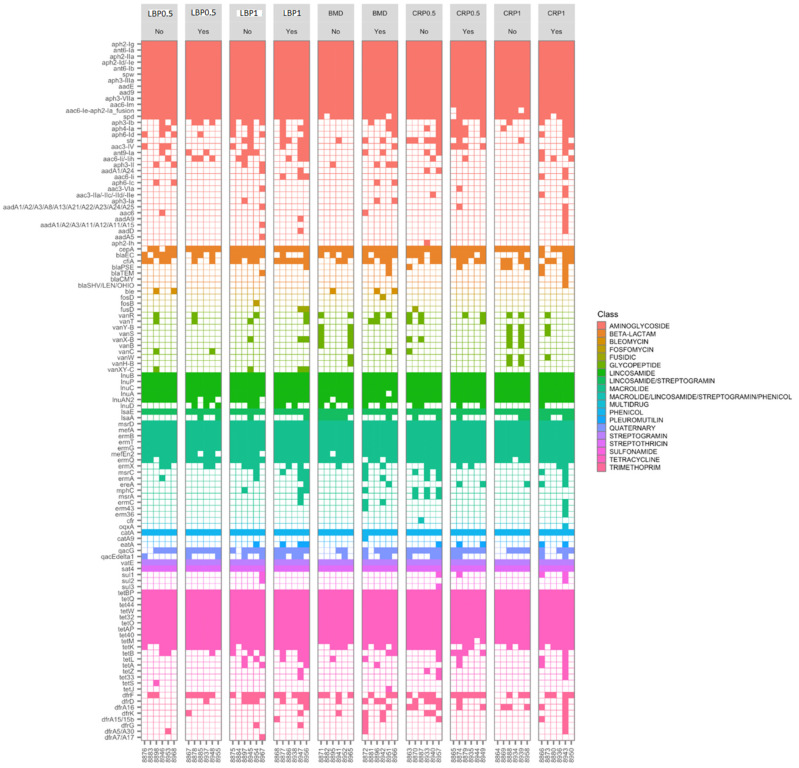
Heatmap of targeted-resistome sequencing showing the presence and absence of specific antimicrobial resistance genes identified. Results are from ceca of 35-day-old birds fed bacitracin (BMD), or cranberry (CRP at 0.5 and 1%) and blueberry (LBP at 0.5 and 1%) pomaces alone or in combination with a multienzyme mixture (ENZ).

All 60 cecal DNA samples from 35-day-old broilers, resulting in a total sequencing depth of c.a. 12.9 ± 3.7 million reads, with an average read survival (post-host removal) rate and on-target rate of 85 ± 9% and 38 ± 10%, respectively. About 116 antimicrobial resistance genes (AGRs) were detected with the genes conferring resistance to aminoglycosides (*n* = 34), tetracyclines (*n* = 17), macrolides (*n* = 16), lincosamides (*n* = 8), and β-lactams (*n* = 7) being the most frequently and abundantly detected (Table 2). The aminoglycoside (*aph2-Ig*, *ant6-Ia*, *aph2-IIa*, *aph2-Id/-Ie*, *ant6-Ib*, *spw*, *aph3-IIIa*, *aadE*, *aad9*, *aph3-VII*, *aac6-Im*, and *aac6-Ie-aph2-Ia_fusion*), tetracycline (*tetBP*, *tetQ*, *tet44*, *tetW*, *tet32*, *tetO*, *tetAP*, *tet40*, and *tetM*), and lincosamide (*lnuA*, *lnuB*, *lnuC*, *lnuAN2*, *lnuP*, and *lsaE*) resistance genes were found in all 60 ceca samples. Of them, 57 (95%), 39 (65%), and 37 (62%) were positive for the β-lactam resistance *cepA*, *blaEC*, and *cfiA* genes, respectively, while *bla*_PSE_, *bla*_TEM_, *bla*_CMY_, and *bla*_SHV_ were detected in 9, 4, 1, and 1 cecal samples, respectively (Figure 6).

**Figure 7:**
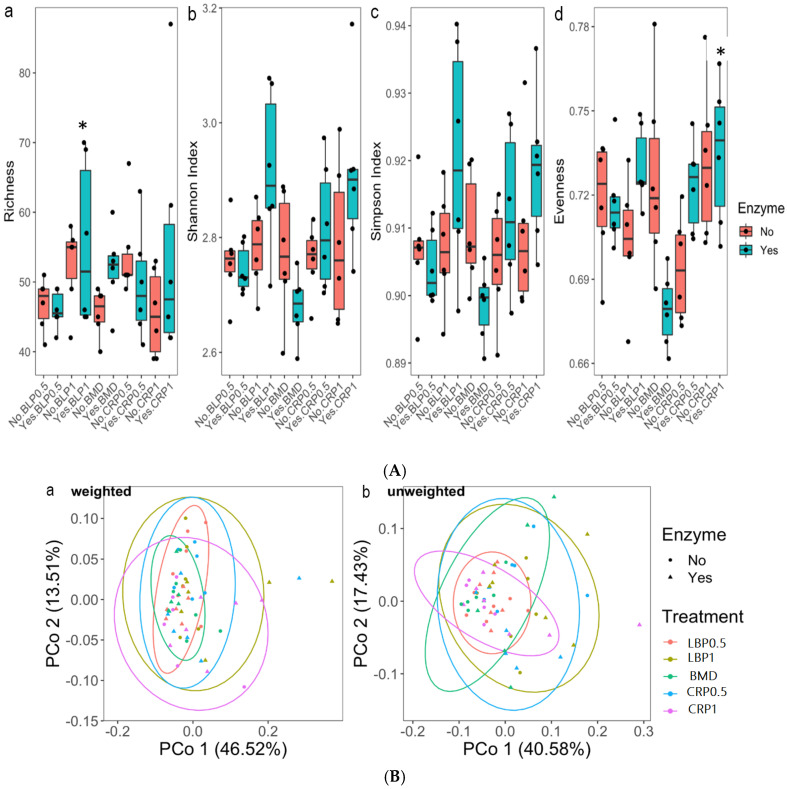
(**A**) Alpha-diversity metrics of ARGs in 35-day-old broiler ceca broiler chickens fed bacitracin (BMD), or cranberry (CRP at 0.5 and 1%) and blueberry (LBP at 0.5 and 1%) pomaces alone or in combination with a multienzyme mixture (ENZ): (**a**) richness, (**b**) Shannon index, (**c**) Simpson index, and (**d**) Evenness. * indicates statistical difference (*p* < 0.05). (**B**) Beta-diversity of total resistance genes (resistome) in 35-day-old broiler chicken fed bacitracin (BMD), or cranberry (CRP at 0.5 and 1%) and blueberry (LBP at 0.5 and 1%) pomaces alone or in combination with a multienzyme mixture (ENZ). The results revealed by the PCoA plots of weighted (**a**) and unweighted (**b**) Bray–Curtis showed clustering based on the studied diets. Significant dissimilarity was not detected between BMD-treated and other groups (weighted only *p* < 0.05). No significant resistome dissimilarity was detected between the non-ENZ and ENZ groups.

The alpha-diversity analysis using the non-parametric statistics (Kruskal–Wallis test) and Post hoc (Dunn’s test) pairwise comparison showed significant differences for Shannon and Evenness (Figure 7A). Differences between BLP1 and BMD and CRP1 and BMD for the enzyme-fed groups were noted (*p* < 0.05). Beta-diversity of the total resistance genes (resistome) revealed by the PCoA plots of weighted and unweighted Bray–Curtis showed dissimilarities between the studied diets. Significant dissimilarity was not detected between the BMD-treated and other groups (weighted only *p* > 0.05). No significant resistome dissimilarity was detected between the non-ENZ and ENZ groups (Figure 7B).

Table 2 shows that in diet, ENZ tended to increase the relative abundance of *athe ph4Ia*, *aac3IV*, *bla*_PSE_, *ereA*, *tetB*, and *dfrA16* genes, whilst significantly (*p <* 0.05) increasing the abundance of *cepA*, *lnuAN2*, *lnuD*, *ermG*, *sat4*, *tetO*, the macrolide *(MACROLIDE)*, and tetracycline (*TETRACYCLINE)* resistance genes. Treatment with CRP0.5 decreased the abundance of *cepA* and *MACROLIDE* but increased the abundance of the *meFEn2* genes. Feeding CRP1 decreased the abundance of *InuD* but increased that of the *tetQ* genes. In the LBP0.5-fed birds, the abundance of *InuA2* genes was low, while LBP1 feeding increased those of tetracycline resistance genes. In contrast, BMD in the diet decreased the abundance of tetracycline resistance *tetBP* and *tetAP* genes. Treatmentwise, the weighted clustering of ARGs in the PCoA chart (Figure 6) indicates the dissimilarity between the BMD-fed and other fed groups for ARGs.

### 3.4. Correlations Between Cecal Microbiota and ARGs

**Figure 8:**
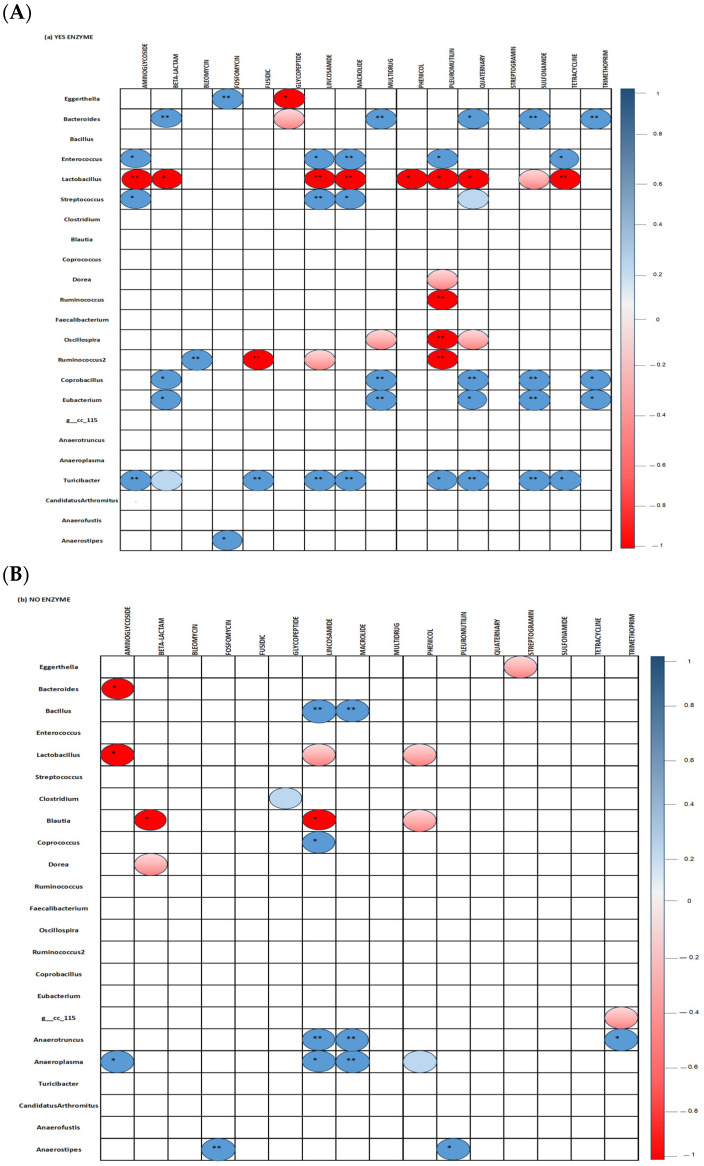
Spearman non-parametric correlations between bacterial genera and antimicrobial resistance genes in the ceca of 35-day-old broiler chickens fed studied diets with (**A**) or without (**B**) a multienzyme mixture. All statically significant correlations are indicated (* *p*< 0.05 and ** *p* < 0.001).

Correlation between bacterial genera and ARG classes in the ceca of 35-day-old broiler fed diets with (Figure 8A) or without (Figure 8B) the studied multienzymes are presented. The inclusion of ENZ in diets resulted in a significant positive correlation (*p* < 0.001) between *Turicibacter* and genes conferring resistance against aminoglycosides, beta-lactams, fusidic, lincosamides, macrolides, quaternary compounds, sulfonamides, pleuromutilin, and tetracyclines (*p* < 0.05). Positive correlations were also found between *Eubacterium* and genes encoding beta-lactam, multidrug, quaternary compound, sulfonamide, and trimethoprim resistance as well as between *Streptococcus* and aminoglycoside, lincosamide, and macrolide resistance genes; *Enterococcus* and aminoglycoside, lincosamide, tetracycline, and macrolide antibiotics resistance genes; *Ruminococcus* and resistant genes against bleomycin; and *Bacteroides* and resistant genes against beta-lactams, multidrugs, quaternary compounds, sulfonamides, and trimethoprim (*p* < 0.05). The inclusion of ENZ in the diets induced significant negative correlations between *Lactobacillus* and genes encoding aminoglycoside, lincosamide, macrolide, phenol, pleuromutilin, quaternary compound, sulphonamide, and tetracycline resistance genes as well as between *Ruminococcus* and fusidic and pleuromutilin resistance genes (*p* < 0.001). In the no-ENZ diets, *Lactobacillus* and *Bacteroides* were negatively (*p* < 0.05) correlated with aminoglycoside resistance genes while *Blautia* was negatively correlated with beta-lactam and lincosamide resistance genes. Moreover, positive correlations were observed between *Bacillus* and *Anaerotruncus* and lincosamide and macrolide resistance genes, as well as between *Anaeroplasma* and genes conferring aminoglycoside, lincosamide, and macrolide resistance (*p* < 0.001).

### 3.5. Metabolomics Profile

**Figure 9:**
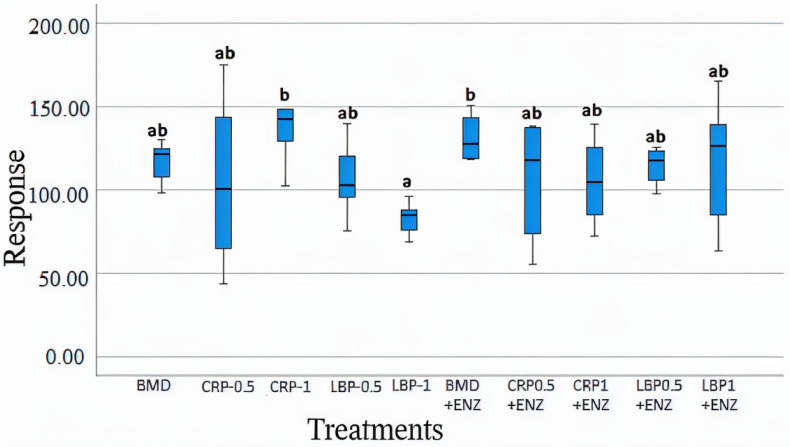
Cecal acetate concentrations in day 21-day-old broiler chicken fed bacitracin (BMD), or cranberry (CRP at 0.5 and 1%) and blueberry (LBP at 0.5 and 1%) pomaces alone or in combination with a multienzyme mixture (ENZ). The relative levels of acetate were determined by comparison to an internal standard of a known concentration. The treatment groups sharing the same letters (a, ab, or b) on the top of boxes indicate no significant differences (*p* > 0.05).

Various chemicals were recognized by different spectra and relative concentrations were estimated by comparison with internal standards; however, we focused on the SCFAs. At D21, the CRP1 alone and BMD + ENZ groups showed significantly higher concentrations of acetate (*p* < 0.05) than the LBP1 group (Figure 9). There were no significant differences between the ten treatment groups for butyrate, glutamate, valerate, and propionate concentrations (*p* > 0.05). The concentrations of acetate, butyrate, propionate, and valerate were all significantly higher at D35 than at D21. Overall, the addition of fruit pomaces and/or the multienzyme mixture showed no significant effects on most measured chicken cecal metabolites compared to the standard BMD treatment (*p* < 0.05).

## 4. Discussion

The use of fruit by-products (pomaces) has been the subject of research in human health and is gaining attraction in animal production. This is due to the phytochemical composition of these products and their potential beneficial effects on gut health. The present study investigated the impacts of providing broilers with diets containing American cranberry and low-bush wild blueberry pomaces, without or with exogenous feed enzymes, on the cecal microbiota, metabolites, and the resistome. Effects of feeding diets containing berry pomaces and enzyme combinations on broiler growth performance and plasma metabolite profile have been previously reported [29]. Similarly, studies on the use of fruit by-products such as those from citrus, grapes, and pomegranate in poultry diets have led to improved feed efficiency, reduced abdominal fat, reduced oxidative stress, improved immune response, and modulated intestinal microbiota [47,48].

Increased cecal coliform counts from LBP diets supplemented with ENZ and a decrease in coliforms with ENZ-supplemented CRP0.5 diets demonstrated the different effects that these products have on microbial populations. Wild blueberry pomace supplementation and basal diet-fed broilers raised on pasture showed decreased Gram-negative bacteria counts according to Islam et al. [49]. This disagreement between the latter results and those from our present study is probably first due to the pasture-raised vs. conventional barn-raised birds in our study. Additionally, the use of the enzymes in our study could impact microbial dynamics by way of the enzymes breaking down fiber fractions to make them accessible to cecal and colonic bacteria for the bacteria’s own metabolic processes, hence increasing their population [50]. The biological significance of berry pomace effects on coliform counts related to gut health needs to be determined in future research. Previous reporting on the characterization of the pomaces used in this study showed higher anthocyanins and tannins in LBP compared to CRP and higher manganese in LBP compared to CRP [51]. Manganese is an important trace element involved in bacterial survival and fitness through the regulation of the metabolism of proteins and carbohydrates, and DNA replication [52]. This high level of manganese in LBP, we posit, could contribute to the higher coliform counts observed. Research investigating the effects of acacia fiber on gut colonization in mice showed that fiber fractions in diets decreased *E. coli* colonization by acting as prebiotics [53]. In the present study, gut health was not investigated; our hypothesis was that enzymes would break down fiber which could result in an increase in nutrient availability for beneficial cecal bacteria, leading to the competitive inhibition of colonization by pathogenic bacteria [54].

The culture-independent characterization of these cecal microbiota showed that in all the treatments, more than 95% of the bacteria were from the Firmicutes and Bacteroidetes phyla. Extensive work by Wei, Morrison [55] in chickens has detailed that Firmicutes, Bacteroidetes, and Proteobacteria are the most predominant bacteria found in the poultry gut. In the current study, we observed similar bacterial phylum profiles in the ceca of 21- and 35-day-old broilers. Bacteria belonging to these phyla are involved in influencing nitrogen recycling through uric acid to produce essential amino acids and digestion of non-starch polysaccharides to produce short-chain fatty acids [56]. Age effects were apparent as shown by the decrease in Proteobacteria in all the groups except birds fed LBP1 + ENZ at D21. This effect of LBP1 + ENZ compared to BMD to increase Proteobacteria is important and critical, knowing that most pathogenic bacteria from *Enterobacteriaceae* belong to this phylum. Bacteria genera from *Enterobacteriaceae*, including *Salmonella*, *Escherichia*, and *Shigella*, have been implicated in global economic losses in poultry production [57]. This outcome, together with the increased abundance of *Peptostreptococcaceae* and *Streptococcaceae* (two Gram-positive bacteria) in day 35-day-old birds compared to 21-day-old ones, needs to be determined. This increase in *Streptococcaceae* could be attributable to tannins in blueberry pomaces. Tannins have the effect of chelating iron, which creates unfavorable conditions for other bacteria than *Lactobacillus* [19]. Additionally, *Streptococcaceae* possess a β-glucosidase enzyme that is able to metabolize the phenolic anthocyanins of blueberry into metabolites such as *p*-coumaric acid and benzoic acid that can be utilized by the bacteria [49]. *Peptostreptococcaceae* is a known commensal of the chicken intestine, which is thought to assist in the maintenance of gut homeostasis [58], and therefore, an increase in its population portends a beneficial effect of the berry products regardless of enzyme usage in the diets. Other bacterial genera such as *Faecalibacterium*, *Ruminococcus*, and *Coprobacillus* were abundant in the cecal contents of the birds fed cranberry products; though not statistically significant, their presence is important as these bacteria could aid in the competitive exclusion of pathogenic bacteria from the gut and act as anti-inflammatory bacteria such as *F. prausnitzii*, for example [59]. Except for CRP0.5 fed alone, berry pomace alone or in combination with ENZ reduced the Log FC abundances of *Proteobacteria* (*Enterobacteriaceae*). This is an interesting finding especially when observed from the angle of ENZ as no linear dose effects were found in the absence of ENZ. Enzyme mechanics have been described by Ravindran [60], Slominski [61], and Meng et al. [23], reporting additive or sub-additive effects of enzymes on certain feed ingredients independently of factors, including the blend of substrates and enzyme mixture available to act on the substrates.

The resistome analysis and results from this study demonstrated the impact of pomaces in either increasing or decreasing the prevalence and relative abundances of some ARGs. In a study by Yang et al., CRP-supplemented diets were shown to reduce the abundance of ARGs when compared to basal and BMD diets, especially genes related to efflux pumps and resistance to phenolic compounds were affected [62]. In our present study, blueberry and cranberry pomaces increased the population of *Bacteroides fragilis* and *Bacteroides ovatus*. *Bacteroides* spp are normal Gram-negative, anaerobic bacilli of the human and animal gastrointestinal tract, some of which can occasionally cause human invasive infections. Despite that, *Bacteroidetes* could benefit their host by excluding potential pathogens from colonizing the gut, and some of them, including *B. fragilis*, can be pathogenic and harbor ARGs such as the *cfiA* gene encoding a metallo-β-lactamase of Ambler’s class B or the *cepA* gene encoding a β-lactamase of Ambler’s class A [63]. Other ARGs including tetracycline resistance *tetX* and *tetQ* genes have been identified in *B. fragilis* isolates [64] and this could be postulated as the reason for the increase in the tetracycline-resistant genes observed in our study. In *B. fragilis*, Insertion Sequence (IS) elements have been suggested to play a key role in the expression of diverse ARGs such as *erm* genes [64]. However, in our study, it is worth pointing out that the *Lactobacillus* and *Bacteroides* genera, whose populations were increased by CRP diets, correlated negatively with aminoglycosides resistant genes, which provides further evidence of the importance of these bacteria in their utilization as probiotics to enrich the gut microbiome of poultry and humans to help in combating disease states [26,65]. In-diet CRP reduced the abundance of *cepA*, *MACROLIDE*, and *InuD*, which correlated to the reduced abundance of bacteria in the Proteobacteria phylum.

Diet compositions and nutrient availability are important factors in modulating gut bacterial communities that could harbor ARGs. Thus, dietary interventions allowing a buildup of bacterial communities with low ARGs are critical in fighting against AMR. Accordingly, it has been reported that the aminoglycoside resistance *aph3-dprime* gene correlated negatively with total calories and soluble fiber intake and that low ARGs correlated with the consumption of fiber in diets [66]. The importance of fiber and polyphenolic components in CRP and LBP for broilers has been previously reported [28]. The studied pomaces are known to be rich in fibers, and the multienzyme in the present study included galactanase, protease, β-mannanase, β-glucanase, xylanase, α-amylase, and cellulase which were prepared based on pomace compositions described in previously published works (Islam et al. [20,49] and Ross et al. [51]). These enzymes are known to solubilize and degrade the antinutritive factors non-starch polysaccharides (fibers) which could lead to the release of starch, fats, proteins, and carbohydrates to improve nutrient availability and fiber solubilization for their effective utilization by birds. In-diet enzymes resulted in 36 positive and 19 negative correlations between the cecal bacterial genera and ARGs compared to 13 positive and 10 negative correlations found in the no-enzyme diet. The results from the present study indicated that the increase in *Bacteroides* abundance decreased those of aminoglycoside genes when the enzyme was included in the feed. This could be explained by the fact that Bacteroidetes could exclude potential bacteria harboring aminoglycoside resistance genes in the gut as stated above. These results indicate potential relationships between the use of enzymes on ARGs in broiler ceca which could be related to the diet fiber content. The effect of ENZ in tending to or significantly increasing the numbers of specific ARGs needs a deeper understanding to establish further correlations between the higher population of *Streptococcus*, *Eubacterium*, *Coprobacillus*, and *Turicibacter* and the ARGs mentioned in the Section 3. To be specific, the questions emerging would be how the physico-chemical enzymatic process of fiber degradation leads to the selection of bacterial species containing ARGs.

In broiler chickens, the gut microbiota community and structure change from hatching to about 35 days (slaughter stage) [67]. These changes influence the gut metabolite profile whose understanding could offer insight into health status and energy metabolism efficiency [67]. Studies by Colombino et al. showed that the ileal short-chain fatty acids were higher in pomace diets (apple, strawberry, and black currant) compared to the control diets [68]. Our metabolomic results showed that in birds grown under standard conditions, acetate, propionate, butyrate, and valerate increase from the first day of life, stabilizing around 21 days of age in alignment with the maturation of the microbiota [69]. This could explain partially the little or no significant differences observed between treatments for the majority of the metabolites estimated in the present study. Moreover, there was a general but not significant increase in acetate, butyrate, propionate, and valerate at D35 compared to D21, which could be explained by the effect of age, with older birds having a stable microbiota population, which aids in breaking down the fiber matrix of pomace diets [23]. High intra-variabilities between the analyzed samples could also, in part, explain the non-statistical differences observed between the treatments.

## 5. Conclusions

Berry products have shown their impact in positively modulating the gut microbiota of broilers when compared to bacitracin, especially in the early life of broilers when pathogenic bacteria have far-reaching consequences in terms of mortalities, morbidities, and economic losses. The two berries have shown different effects at different ages on coliforms, microbiota, and resistome, with our study showing new findings about the correlations between ARGs and berry pomace feeding in the presence or absence of fiber-degrading feed enzymes. We surmise that there could be probable causational aspects of pomaces modulating cecal bacterial metabolism and subsequent ARGs by providing tannic acid as a metabolizable substrate, especially in enzyme-treated diets with wild blueberry pomace. A deeper look at individual bacterial isolates and their ARGs would give more informed corroborative evidence of the resistome and microbiota profile, in turn assisting in assessing the cost and benefits, and balancing between the use of feed enzymes and fruit pomaces in poultry feed formulations to facilitate nutrient breakdown (and utilization) and their possible influence on ARGs. Further studies should explore the balance between the benefits and detriments accrued by the use of cranberry pomace and blueberry pomace in relation to the resistome and microbiota so that an equilibrium can be achieved.

## Figures and Tables

**Table 1:** Effects of feeding broiler chickens with diet without or with a multienzyme mixture on the concentration of coliforms in the cecal digesta.

	No Enzyme					Plus Enzyme								
Day	* Treatment					Treatment						*p*-Values		
	BMD	LBP0.5	LBP1	CRP0.5	CRP1	BMD	LBP0.5	LBP1	CRP0.5	CRP1	SEM	ENZ	Treat *	ENZ * Treat
21	1.9 ^a^	0.6 ^c^	0.56 ^c^	1.61 ^ab^	1.38 ^b^	1.52 ^b^	1.67 ^ab^	1.81 ^a^	1.44 ^b^	1.57 ^b^	0.336	0.067	0.378	0.054
35	2.38	2.71	2.62	1.86	2.38	2.24	1.76	2.22	1.27	2.33	0.234	0.005	0.003	0.287

Log_10_-transformed data * dietary treatment (Treat) consisted of diet plus BMD (55 ppm bacitracin methylene disalicylate), CRP0.5 (cranberry pomace at 0.5% dietary inclusion level), CRP1 (cranberry pomace dietary at 1% inclusion level), LBP0.5 (blueberry pomace at 0.5% dietary inclusion level), and LBP1 (blueberry pomace at 1% dietary inclusion level). Data represents SEM of seven replicates/treatments (*n* = 7 pens of 2 birds/pen) arranged in a completely randomized block design. *p*-value obtained by ANOVA. ^abc^ means in the same row with different superscripts have significant differences (*p* < 0.05) while means with the same superscript letter are not significantly different.

**Table 2:** Abundances of significant antimicrobial resistant gene affected by dietary bacitracin or berry pomaces without or with a multienzyme mixture in 35-day-old broiler chickens.

Gene	Enzyme				Treatment *					*p*-Values		
	No	Plus	SEM	BMD	CRP0.5	CRP1	LBP0.5	LBP1	SEM	Enzyme	Treatment	Enz * Treat
*cepA*	3.2 ^b^	3.3 ^a^	2.52	3.3 ^a^	3.1 ^b^	3.3 ^a^	3.3 ^a^	3.2 ^ab^	2.72	0.005	0.031	0.994
*lnuAN2*	3.4 ^b^	3.6 ^a^	2.82	3.4 ^bc^	3.5 ^b^	3.8 ^a^	3.2 ^d^	3.4 ^bc^	3.02	0.023	0.030	0.866
*lnuD*	1.8 ^b^	2.1 ^a^	1.55	2.0 ^bc^	2.2 ^a^	1.3 ^d^	1.7 ^c^	2.1 ^ab^	1.75	0.018	0.050	0.953
*ermG*	5.0	5.0	3.94	5.1	5.0	5.0	5.0	5.1	4.14	0.058	0.104	0.968
*mefEn2*	3.6 ^b^	3.9 ^a^	3.06	3.6 ^bc^	3.8 ^ab^	4.0 ^a^	3.4 ^d^	3.6 ^bc^	3.26	0.022	0.032	0.858
*MACROLIDE*	4.4 ^b^	4.6 ^a^	3.24	4.4 ^a^	4.3 ^b^	4.4 ^a^	4.4 ^a^	4.4 ^a^	3.44	0.012	0.028	0.997
*NBMICROLIDE*	0.9	0.9	0.51	0.9	0.9	0.9	0.9	0.9	−0.32	0.536	0.731	0.051
*sat4*	4.7 ^a^	4.6 ^b^	3.50	4.7	4.6	4.6	4.7	4.7	3.70	0.020	0.244	0.960
*tetBP*	4.2	4.3	3.56	3.8 ^d^	4.3 ^b^	4.1 ^bc^	4.3 ^b^	4.5 ^a^	3.76	0.880	0.049	0.810
*tetQ*	3.9 ^b^	4.1 ^a^	3.31	4.0 ^b^	4.0 ^b^	4.2 ^a^	4.0 ^b^	4.0 ^b^	3.51	0.006	0.024	0.976
*tetO*	4.6	4.5	3.41	4.6	4.5	4.5	4.5	4.6	3.61	0.098	0.155	0.922
*tetAP*	4.0	3.9	3.22	3.5 ^cd^	4.0 ^ab^	3.9 ^bc^	3.6 ^c^	4.2 ^a^	3.42	0.480	0.033	0.831
*TETRACYCLINE*	4.4	4.4	3.21	4.4 ^b^	4.4 ^b^	4.4 ^b^	4.4 ^b^	4.5 ^a^	3.41	0.088	0.003	0.999

Log_10_-transformed represents means ± SEM of six replicates/treatments (*n* = 6 pens of 2 birds/pen) arranged in a completely randomized block design. *p*-value obtained by ANOVA. * dietary treatment: BMD (55 ppm bacitracin methylene disalicylate), CRP0.5 (cranberry pomace at 0.5% dietary inclusion level), CRP1 (cranberry pomace dietary at 1% inclusion level), LBP0.5 (blueberry pomace at 0.5% dietary inclusion level), and LBP1 (blueberry pomace at 1% dietary inclusion level); ^abcd^ means in the same row with different superscripts have significant differences (*p* < 0.05) while means with the same superscript letter are not significantly different.

## Data Availability

The sequence reads data of the 16S rRNA and shotgun metagenome of this study has been submitted to the Sequence Read Archive (SRA) database of the National Center for Biotechnology Information as FASTQ files under the Bioproject accession number PRJNA1048927.
